# Nutrient Composition Comparison between the Low Saturated Fat Swank Diet for Multiple Sclerosis and Healthy U.S.-Style Eating Pattern

**DOI:** 10.3390/nu11030616

**Published:** 2019-03-13

**Authors:** Catherine A. Chenard, Linda M. Rubenstein, Linda G. Snetselaar, Terry L. Wahls

**Affiliations:** 1 Department of Internal Medicine, Carver College of Medicine, University of Iowa, Iowa City, IA 52242, USA; terry-wahls@uiowa.edu; 2 Department of Epidemiology, College of Public Health, University of Iowa, Iowa City, IA 52242, USA; linda-rubenstein@uiowa.edu (L.M.R.); linda-snetselaar@uiowa.edu (L.G.S.)

**Keywords:** low saturated fat diet, exemplary menus, nutritional adequacy, nutrient density, HEI-2015, AHEI-2010, multiple sclerosis, Swank diet

## Abstract

Multiple sclerosis (MS) is an incurable degenerative disease that attacks the central nervous system. Roy Swank proposed a low saturated fat diet to treat MS around 1950 and showed delayed disease progression in his patients. However, there is insufficient evidence to recommend this diet for MS and default dietary recommendations are the Dietary Guidelines for Americans (DGA). This study assessed the nutritional adequacy of seven-day menus developed by Swank and their compliance with the DGA; menus were modeled for comparison with the DGA Healthy US-Style Eating Pattern (HEP) for males and females 31–50 years. Swank recommended dietary supplements corrected menu shortfalls in vitamins D, E, calcium, folate and iron but not dietary fiber, potassium and choline. Healthy Eating Index-2015 score for Swank menus (93.2/100) indicated good compliance with the DGA. Nutritional adequacy of the Swank modeled diet was similar to HEP for 17 vitamins and minerals (Mean Adequacy Ratios ≥94%) with similar shortfall nutrients except magnesium (HEP males) and dietary fiber (Swank males). Alternate Healthy Eating Index-2010 scores for Swank male (90/110) and female (88/110) model diets were similar to HEP. Swank menus have similar nutritional adequacy as HEP. Inclusion of foods high in dietary fiber, potassium and choline may be advised as well as selection of foods to reduce sodium below the Tolerable Upper Intake Level.

## 1. Introduction

Multiple sclerosis (MS) is an incurable immune-mediated, inflammatory disease that attacks the central nervous system. Persons with MS (pwMS) may experience visual disturbances, cognitive and emotional changes, movement and balance difficulties, bowel and bladder dysfunction, pain and fatigue. The symptoms can wax and wane over time as myelin and axons are damaged and then partially repaired. As the disease progresses, the accumulated damage may lead to greater disability, but the disease course is unpredictable [1,2]. Symptoms consistent with MS were reported as early as the 1300 or 1400s but it was not until the pathology was associated with the symptoms in the 1800s [3,4] that MS as a distinct condition, separate from diseases such as Parkinson’s disease, emerged [3,5]. Eventually diagnostic methods and access to trained neurologists improved [3]. By 1950 MS was the most common neurological disease in the United States (US) [5] but there were no effective treatments [4].

Beginning in 1948 neurologist Roy Swank, MD, PhD began investigating the epidemiology of MS and proposed a low saturated fat dietary treatment [6] based on data suggesting that a higher intake of saturated animal fats were associated with higher incidence of MS [7,8]. Swank placed his patients on this diet and followed them for 50 years [9], publishing a series of reports [10,11,12,13,14,15,16,17,18,19]. His main findings were a reduction in the frequency and severity of relapses when patients consumed ≤20 g saturated fat/day [9,10,11,12,13,14]. These patients exhibited less disability and lower mortality [17] especially when the diet was started early in the disease course [10,11,15,17]. However, his study has been criticized for comparing good versus poor diet adherents which biases the data towards positive results, for lacking a control group, blinded assessors, brain imaging data [20,21] and standardized dietary intake assessment and for missing data that is not missing at random [22].

Swank did not believe fat caused MS but that a high intake might contribute to a more rapid onset of the disease progression in susceptible individuals [7,23]. He thought this may be the result of the obstruction of small blood vessels due to increased clustering of chylomicrons after consumption of a high fat meal [10,23,24,25,26,27]. Various vessel-based theories of MS have been suggested since 1863 and continue today with the chronic cerebrospinal venous insufficiency theory [28] and hypotheses of cholesterol and disordered lipid metabolism [29,30]. Total cholesterol [31] and low density lipoproteins [32] have been positively associated with disability. Lipids were not associated with risk of relapse in 141 Australian pwMS [33] but energy intake from fat and saturated fat were associated with relapse in a pediatric MS study [34,35].

Swank published a book that summarized his findings and provided practical advice, recipes and seven-day menus to assist pwMS in following his diet [36]. He died in 2008 [6] but his diet continues to be promoted by the Swank MS Foundation [37]. PwMS are still following this diet today [38,39,40,41]. Dietary guidelines for the Swank diet are shown in Table 1.

In the 70 years since Swank developed his diet, various other dietary regimens have been proposed to treat MS including the plant-based low-fat McDougall [42], Mediterranean [43,44], ketogenic [45], energy restricted/fasting [46,47,48] and modified Paleolithic (Paleo) Wahls™ [49,50,51,52,53]. However, more research and better designed studies are needed to determine the benefits and risks of the Swank or any other diet for pwMS [54,55]. To help address this research gap, dietary intervention studies are underway that investigate intermittent fasting (NCT03539094), dietary salt and immune function (NCT02282878), ketogenic (NCT03718247) and low fat (<20% energy) diets (NCT03322982), ketogenic versus intermittent fasting versus vegetarian diets (NCT03508414), activity and balanced eating (NCT03808545), a low glycemic load diet administered via internet coaching (NCT03372187) and the effect of a gluten-free diet on blood brain barrier permeability (NCT03451955). Until there is sufficient scientific evidence to determine which diet(s) are beneficial for MS, the National MS Society (NMSS) [56,57] encourages pwMS to follow healthy eating recommendations such as the Dietary Guidelines for Americans (DGA) [58] and those promoted by the American Heart Association [59] and American Cancer Society [60].

One clinical trial currently underway is investigating the effect of the Swank diet on MS-related fatigue [61,62] but there are no reports of the Swank diet’s nutritional adequacy or compliance with the DGA. Swank considered his diet to be ‘healthy’ because of the reduction in fat and ‘junk’ food [36]. The NMSS [56] does not believe the Swank diet would cause nutrient deficiencies but one study found a low intake of vitamins A, C, E and folate in the 24 h recalls of two pwMS following the diet [39]. In addition, individuals consuming diets low in fat may be at risk for low intake of some nutrients [63] such as vitamin E and linoleic acid [64].

Therefore, the purpose of this report is to assess the nutritional adequacy of the Swank diet by comparing nutrient levels to the Dietary Reference Intakes (DRI) [65,66] for adult men and women. The diet’s adherence to the DGA, the current default dietary guidance, will be assessed using the Healthy Eating Index 2015 (HEI-2015) [67]. Scores for the Alternate Healthy Eating Index 2010 (AHEI-2010), which have been associated with risk of chronic diseases such as coronary heart disease and diabetes [68], will also be calculated. The Swank diet’s nutritional adequacy, HEI-2015 and AHEI-2010 scores will be compared to those for the Healthy US-Style Eating Pattern (HEP) [69] recommended by the DGA.

## 2. Materials and Methods

### 2.1. Study Overview

This study was considered exempt by the University of Iowa Institutional Review Board. It reports the calculated nutrient composition, nutritional adequacy, and HEI-2015 scores of seven-day menus developed by Roy Swank [36]. Nutritional adequacy and HEI-2015 scores for the HEP were obtained from published data [70,71]; AHEI-2010 scores were calculated using publicly available food and nutrient data used to develop the HEP [69,70,72]. Nutrient composition of the Swank seven-day menus was modeled using United States Department of Agriculture (USDA) nutrient profiles to allow for comparison with the nutritional adequacy of the 2015–2020 DGA HEP and calculation of the AHEI-2010.

### 2.2. Nutritional Adequacy of Seven-Day Swank Menus

Seven-day Swank menus and associated recipes were used as published in *The Multiple Sclerosis Diet Book* [36] except that refined grains (i.e., rice, crackers, bread, pasta and waffles) were replaced with whole grain versions to be consistent with current Swank MS Foundation guidelines that encourage use of whole grains [37]. Swank menus are described in Supplementary Table S1.

Nutrient composition of Swank menus was calculated by a Registered Dietitian using Nutrition Data System for Research (NDSR) software version 2017 (Nutrition Coordinating Center (NCC), University of Minnesota, Minneapolis, MN, USA) [73]. To ensure consistent entry of ambiguous recipe and menu details, these data entry rules were followed. 1. When amounts were listed as a range, the average was used (e.g., 11 nuts were entered for “10–12 peanuts, almonds, cashews” for Sunday snack). 2. When multiple ingredients were listed as an option, all items were used in equal proportions (e.g., 1/3 each peanuts, almonds and cashews entered for Sunday snack) except for Swank day 4 dinner when a full serving of rice was selected instead of a half serving each of rice and potato because rice is a more typical side dish for the oriental entrée at this meal. 3. When nuts were an optional ingredient in a recipe, half the recommended amount was used so as to provide an average of the two options for zero nuts or the full amount. 4. No salt was added in cooking unless specified in the recipe; one dash per serving was used when directions specified salting to taste. The NDSR salt default was used for commercial foods. 5. Frozen fruit was assumed to be unsweetened. 6. Skin was assumed to be retained on fruits and vegetables unless recipe or menu stated otherwise. 7. When food details or amounts were not specified, they were determined using a. Nutrition software data-entry rules (e.g., ½ cup milk per 1 cup cereal used for amount of milk in “cold cereal and skim milk” for Wednesday breakfast) or defaults (e.g., Wednesday breakfast “cold cereal” entered as cereal, ready-to-eat, unknown type which defaults to Cheerios^®^) b. Amounts for 1 cup-equivalent fruit or vegetable (e.g., unknown amount of “juice” on Sunday breakfast entered as 1 cup) or 1 grain-equivalent (e.g., unknown amount of “rice” on Sunday dinner entered as ½ cup cooked) c. Based on representative foods consumed in the US [72] (e.g., oil, fresh fruit and fruit juice types not specified) d. Professional judgment (e.g., selection of NDSR pan fried entry for “fried fish” on Wednesday dinner and NDSR option for green salad with tomatoes and/or carrots and mixed greens for Tuesday dinner “assorted green salad”). 8. The optional unsweetened coffee and tea on the menus were not entered.

Nutrient contribution of dietary supplements was calculated separately from the menus. The NDSR default multivitamin/mineral for the appropriate sex and age was selected for the Swank multivitamin based on the DRI age and sex category being evaluated.

Food group servings were assigned using the NCC Food Group Serving Count System. Recipe food group servings were assigned based on recipe ingredients. Changes were manually made to food group assignment and serving counts for green leaf lettuce to match current guidelines [74].

Individuals use menus and meal patterns to plan their food intake, therefore, mean nutrient composition of the seven-day menus was compared to the Recommended Dietary Allowance (RDA) or Adequate Intake (AI) instead of the Estimated Average Requirement [66,75]. Nutritional adequacy of menus with and without dietary supplements was evaluated using appropriate RDA, AI and Tolerable Upper Intake Levels (UL) for males and females aged 19 to 70+ years [65] by computing the menu average as percentages of the RDA, AI or UL. Prior to this calculation, menu nutrient values were proportionately increased or decreased to produce menus with energy levels appropriate for the varying needs of adult males and females [76].

The average percent RDA for 17 vitamins and minerals (vitamins A, C, D, E, B1, B2, B3, B6, folate and B12, calcium, copper, iron, magnesium, phosphorus, selenium and zinc) was calculated for each age/sex group. A Mean Adequacy Ratio (MAR) [77] (1) was computed using these percentages to provide a score summarizing the overall adequacy of the menus; RDA percentages were truncated at 100% so an excess of one nutrient would not obscure the deficiency of another.

Mean Adequacy Ratio (MAR), % = ∑percent RDA truncated at 100%/number of nutrients.(1)

### 2.3. Nutritional Adequacy of Swank Diet and HEP Using Food Pattern Modeling

The Healthy US-Style Eating Pattern (HEP) [69] included in the DGA [58] consists of a recommended number of servings of food groups (e.g., fruits, vegetables, protein foods, grains, dairy, oils) to create nutritionally adequate diets at various energy levels. The USDA modeled the nutritional adequacy of the HEP [70] using food composition data obtained from the USDA Food Group nutrient profiles [78,79]. The USDA Food Group nutrient profiles [79] were calculated using foods reported in the 2009–10 National Health and Nutrition Examination Survey (NHANES) [72] and nutrient composition for forms of the foods that were low in fat, added sodium and sugar [78,80]. Folate values for the nutrient profiles were not publicly reported but the authors requested them from the USDA [81] for use in this investigation. This report examines the nutritional adequacy of the 1800 and 2200 kcal (7531 and 9205 kJ, respectively) HEP diets which are suitable for females and males 31–50 years, respectively.

Swank menus were modeled similar to the HEP by using the average food group servings from the seven-day Swank menus and the nutrient composition of the USDA Food Group nutrient profiles [79]. Because Swank only provided one set of seven-day menus which did not match the target 1800 and 2200 kcals (7531 and 9205 kJ, respectively), the nutrient composition was proportionately increased to these energy levels prior to comparing to the DRI for males and females 31–50 years. Using the USDA Food Group nutrient profiles to calculate the nutrient composition of the Swank diet allowed for direct comparison with the nutritional adequacy of the DGA HEP which was modeled using these same nutrient profiles [78]. Using the Food Group nutrient profiles also provided an estimate of the nutritional adequacy for the Swank diet as if typical foods consumed in the US had been used to create the menus rather than the foods selected by Swank.

### 2.4. Healthy Eating Index-2015 (HEI-2015) and Alternate Healthy Eating Index-2010 (AHEI-2010)

The Healthy Eating Index-2015 (HEI-2015) [67] is a validated [71] scoring system developed to assess adherence to the 2015–2020 DGA. It is comprised of 13 key food (e.g., total fruit, whole fruit) or nutrient goals (e.g., sodium, added sugar, saturated fat). Four components are to be consumed in moderation and nine for adequacy. The score for each component ranges from zero to five or 10 points. Points are assigned based on the percent energy for that nutrient (e.g., added sugar, saturated fat), the amount of nutrient or food group per 1000 kcals (4184 kilojoules (kJ)) (e.g., mg sodium, cups total fruit) or a nutrient ratio (e.g., fatty acids). Moderation components are scored so that lower amounts receive higher scores. Scores for each component are summed to create a total score indicating overall adherence to the DGA [67]. The maximum score is 100 points indicating perfect adherence.

HEI-2015 total and component scores were calculated for Swank menus using the population ratio method [82]. The scoring system was applied to the ratio of the population’s mean food group (or nutrient) intake to the population’s mean energy intake. HEI-2015 scores for a seven-day 2000 kcal (8368 kJ) sample HEP menu were reported in the literature [71].

The AHEI was developed in 2002 [83,84] to create a score that would predict health outcomes more robustly than the HEI [85,86]. To assess the predictive value of the AHEI scores, it was compared to the HEI scores to determine the strength of risk for development of chronic diseases; however, the HEI was developed to assess adherence to the *Food Guide Pyramid*, the US dietary guidance that was current in 1992 [87], not to predict disease risk. The AHEI was based on dietary components identified by nutrition researchers as being associated with lower risk of some chronic diseases and was shown to provide stronger protective estimates of disease risk, especially cardiovascular disease, compared to the HEI [83,84]. The AHEI was revised in 2012 (AHEI-2010) after a review of the scientific literature and discussion with other researchers [68]. Lower AHEI-2010 scores have been associated with mortality (all-cause, cardiovascular and cancer) in a cohort of British men and women [88] and predicted inflammation (IL-6) in 126 overweight or obese African American females with osteoarthritis [89].

The AHEI-2010 consists of 11 components (food and nutrients) each scored from zero (worst) to ten (best) points. Six components are for adequacy and five for moderation. Similar to HEI-2015, components that are to be limited in the diet are reverse scored (i.e., higher intakes receive lower scores). Total AHEI-2010 score is calculated by summing scores for each component with total scores ranging from zero to 110 points (best). Scores were calculated using menu modeling data for 1800 kcal (7531 kJ) female and 2200 kcal (9205 kJ) male Swank and HEP diets. Minimum and maximum scores for the sodium component were to be assigned based on the dataset’s lowest and highest decile of sodium intake for males and females but the menu modeling did not provide a sodium distribution; therefore, sodium values for the 10th and 90th percentiles for 20+ year old males and females from the 2007-2008 NHANES were used instead [90,91].

Table S2 compares the HEI-2015 and AHEI-2010 components. Both scoring systems address vegetable, fruit, grain and fat consumption albeit in different ways. However, HEI-2015 considers dairy intake while the AHEI-2010 does not and AHEI-2010 considers alcohol consumption while HEI-2015 does not include it as a separate component. Unlike the HEI-2015 which assess all food and nutrients as % energy, per 1000 kcals or as a nutrient ratio, only two of the 11 AHEI-2010 components are assessed this way (polyunsaturated fatty acids, *trans-*fat); however, three components have sex-specific criteria for assigning minimum and maximum points which would take into consideration differences in number of servings or quantities of nutrients generally associated with greater food intake by adult males (whole grains, sodium, alcohol). A systematic review and meta-analysis of the HEI (HEI, HEI-2005, HEI-2010) and AHEI (AHEI, AHEI-2010) concluded that higher scores on both were associated with reduced risk of all-cause mortality, cardiovascular disease and cancer incidence or mortality, type 2 diabetes and neurodegenerative diseases and with cancer and all-cause mortality in cancer survivors [92].

AHEI-2010 scores for Swank and HEP were calculated using nutrient values from menu modeling. Amounts of fruit, vegetable and protein subgroups for HEP were based on data used to generate the food group nutrient profiles [72]; food subgroup servings for Swank were obtained from NDSR. One ounce-equivalent of whole grains was assumed to provide 16 g whole grain. Although alcohol is permitted on both diets in amounts that are within each diet’s guidelines, the model menus did not include alcoholic beverages.

### 2.5. Data Analysis

HEI-2015 and AHEI-2010 calculations and descriptive statistics were performed by the statistician using SAS 9.4 [93] and Microsoft Excel 2010 [94]. Food group servings and nutrients per 1000 kcals (4184 kJ) were computed from the average of all seven menu days. A radar graph which is recommended for displaying HEI-2015 component scores [67] was prepared to illustrate the dietary patterns. No hypothesis testing was conducted.

## 3. Results

### 3.1. Swank Menu Composition

Food group data in Table 2 are consistent with Swank diet guidelines, although amounts are on average slightly more than the minimum recommended servings of vegetables (2 cups), grains (4 servings) and dairy (2 cups) [37]. The oil quantity (oil and salad dressing) is on the low end of the range Swank recommended and is an amount suitable for sedentary individuals (Table 1) but this amount does not include fat from nuts and fatty fish which are also counted as part of the oil allotment on the Swank diet. Mean saturated fat content (Table 3) for 1719 kcal (7192 kJ) was about half the maximum 15 g allotted (range 5.8 g–11.0 g). The menus have a low to moderate glycemic index, one point above the cutoff for low glycemic [95].

### 3.2. Nutritional Adequacy

#### 3.2.1. Swank Menus

The Swank MAR score was ≥94% for females and ≥97% for males 19 years and older, indicating nearly all the RDAs were met (Table 4). For females, vitamin D and E were below the RDA at all ages examined. Folate and calcium were less than the RDA for females 51–70 and >70 years likely due to the lower energy intake at these ages and greater calcium requirement. Iron was below the RDA for females 19–50 years due to the higher iron requirement for these ages. No nutrients were below the RDA for males 19–30 years, likely a result of the higher energy level. Shortfall nutrients were Vitamin E for males 31–50 and vitamins D and E for males 51–70 and >70 years.

Swank menus met the AI for the nutrients examined except the following: choline for females and males 31 years and older; potassium for females 19 years and older and males for ages 31 years and older. Dietary fiber was low for males 31–50 years, likely due to the reduced energy level at that age compared to 19–30 years. No nutrients were below the AI for males 19–30 years, likely due to the higher energy level.

Swank menus exceeded the sodium UL for women 19–50 years and men at all ages. Sodium levels were below the UL for females 51–70 and >70 years, likely due to the lower 1600 kcal (6694 kJ) energy level for these ages.

Menus were within the Acceptable Macronutrient Distribution (AMDR) [96] percentages for protein, fat and carbohydrate (Table 3) with total fat at the lower end of the range (20–35% energy). Menus provided <10% energy from added sugars and <10% energy from saturated fat [58] as recommended by the DGA and <7% energy from saturated fat recommended by the National Lipid Association for reducing serum cholesterol [97] and <5–6% energy recommended by the American Heart Association for individuals who need to reduce their low density lipoprotein cholesterol level [59]. Average saturated fat at all energy levels examined was ≤15.0 g (Table 4), the maximum amount recommended on the Swank diet, but menus would exceed 15.0 g at ≥ 3205 kcal (13410 kJ).

#### 3.2.2. Swank Menus plus Dietary Supplements

Nutrient contribution of dietary supplements is shown in Supplementary Table S3. When the nutrients from supplements were added to the menus, vitamins D, E and folate plus calcium and iron met or exceeded the DRI. The following nutrients remained below the RDA/AI at various age/sex categories: dietary fiber, choline and potassium. Menus did not exceed any additional ULs when supplements were included.

#### 3.2.3. Food Pattern Modeling

The Swank modeled menu macronutrient composition was slightly lower in fat and higher in carbohydrate than HEP (Table 5). Nutritional adequacy of the HEP and Swank model diets were similar with MAR scores ≥94% (Table 5). Nutrients below the RDA in the HEP were identical to Swank except for magnesium which was low in the male HEP but not Swank and dietary fiber which was low in the male Swank model diet but not the HEP. Swank and HEP modeled diets were low in choline and potassium for males and females 31–50 years.

Macronutrient composition of the modeled Swank diet (Table 5) was similar to the Swank menus (Table 3 and Table 4). Nutrients below the RDA for the Swank model diet were similar to the Swank menus except vitamin D was below the male RDA on the model diets (Table 5) but not the menus (Table 4). Swank model diet MAR scores were 1–2 percentage points lower than their menus. AI shortfall nutrients on the Swank model diet were identical to the menus. Sodium did not exceed the UL for the model diet as it did for the menus. The addition of Swank diet-prescribed dietary supplements would meet all RDAs and AIs except for dietary fiber (males), choline and potassium.

### 3.3. HEI-2015 and AHEI-2010

**Figure 1:**
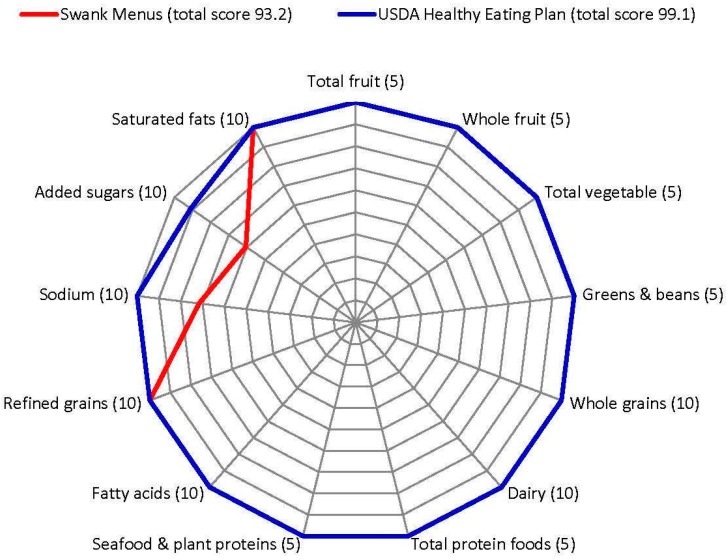
Healthy Eating Index-2015 component scores for seven-day low saturated fat Swank and seven-day USDA Healthy US-Style Eating Pattern menus [71] calculated using the population ratio method. Values in parentheses indicate maximum score for each component. Component scores are plotted on each axis as a percentage of the maximum score and connected with lines. The outermost ring represents a perfect score.

Swank and HEP menus received identical maximum scores for 11/13 HEI-2015 components (Figure 1). However, Swank menus underperformed the HEP for sodium (7.2 versus 10 points, respectively) and added sugars (6.1 versus 9.1 points, respectively). Swank total HEI-2015 score was 5.9 points lower than HEP.

AHEI-2010 scores for Swank 1800 kcal (7531 kJ) female and 2200 kcal (9205 kJ) male diets were 9.1 and 8.8 points higher than HEP, respectively (Table S4). The Swank diet scored higher than HEP for fruit, whole grains, red/processed meat and long chain omega-3 fatty acids (females only). HEP scored higher than Swank for vegetables. Swank and HEP scores for the remaining components differed by ≤1.0 points. The largest difference between Swank and HEP component scores was seen for red/processed meat (5.1 and 6.2 points for female and male, respectively); Swank received a perfect score for this component due to the elimination of red meat as a strategy to reduce saturated fat.

## 4. Discussion

### 4.1. Menu Composition

The Swank menus conformed to the HEP food group recommendations [69] except for differing vegetable and protein subgroup amounts (greater amounts of dark-green vegetables, fish and shellfish, and nuts and seeds, and smaller amounts of red/orange vegetables, beans and peas), fewer dairy servings and slightly more fruit. Nutrition experts generally agree that the inclusion of fruits and vegetables (F/V) are necessary for a healthy diet [98]. Increased vegetable intake has been associated with reduced risk of MS relapse [34] but the potential benefits and harms of grains, dairy and animal/fish protein and fat intake for pwMS are more controversial. An international study of 2087 pwMS found that a ‘healthy’ F/V and fat (e.g., fish, unsaturated oil) intake was associated with better physical and mental health and less disability with fat having the biggest impact. Higher physical and mental health scores were seen when meat or dairy were excluded from the diet but results were not conclusive; grain intake was not assessed [99]. In another study, pwMS consuming higher quality diets defined as high in F/V, whole grains and legumes and low in added sugar and red and processed meat, were associated with less disability and depression; higher intake of whole grains alone was also associated with less disability [100]. Participants in a multimodal healthy lifestyle intervention reported increases in physical and mental health one and three years after diet initiation compared to baseline; the intervention diet included fish, grains and F/V but not meat and dairy, was low in saturated fat (<20 g/day) and included dietary supplements [101]. A 12 month randomized trial investigating a vegetarian (no meat, fish, eggs, dairy or vegetable oils) low (14%) fat diet compared to a usual diet (~40% fat) found improvements in fatigue, weight and blood lipids but no change in disease activity [42].

### 4.2. Nutritional Adequacy

The Swank diet was expected to meet all nutrient requirements [56] but the menus and model diet were low in eight nutrients: vitamins D and E, choline and potassium for most age/sex groups, iron (females 19–50 years), folate (marginally, females ≥51 years), calcium (females ≥51 years) and dietary fiber (males 31–50 years). The low levels of vitamins E and folate were consistent with the low intake of two pwMS following the Swank diet reported in one study [39]. Diet-prescribed supplementation with vitamin C (Table 1) is not needed to meet the RDA but the cod liver oil, multivitamin/mineral and vitamin E supplements would meet all diet shortfalls except for choline, potassium and dietary fiber. Choline is a phospholipid precursor and required for generation of cell membranes and myelin [102,103,104]. It is also involved in the synthesis of acetylcholine, a neurotransmitter [105]. Choline has been identified as a key nutrient which if in insufficient supply would increase the risk of accelerated aging and neurodegeneration [106,107]. Eggs are a top choline source, but only limited amounts can be consumed on the Swank diet due to their high saturated fat content. Additional choline sources include chicken liver, beans/peas, dark-green vegetables or soy products [79] as well as meat, fish, poultry, dairy and peanuts [108]. Potassium is a nutrient of public health concern [58] so individuals following this diet may be advised to select foods high in this nutrient, such as sources in the DGA Appendix 10 [58]. Potassium is important in MS because it may block the potentially adverse effects of sodium [109,110,111].

The dietary fiber intake based on the Swank menus and model diet was slightly lower than HEP and did not meet the AI for males 31–50 years. There is increased recognition that microbes in the gut influence immune cell function in MS and that diet, especially fiber, impacts the gut microbiome [112,113,114]. As specific foodstuffs are digested, the gut microbiome will create metabolites which may be absorbed into the blood stream and favorably impact both systemic and central nervous system (CNS) inflammation [115,116]. Studies are needed to identify the impact of dietary components on gut microbiome and MS clinical course. One trial [61,62] is evaluating changes in gut microbiome, fatigue and quality of life among pwMS randomized to the Swank diet. In addition, dietary fiber is a nutrient of public health concern [58] so ensuring adequate intake is advised.

The Swank diet recommends sufficient energy to achieve and maintain a healthy body weight. Menus used in this report were adjusted to provide appropriate energy levels for sedentary individuals. Swank reported individuals following his diet lost weight because of low energy intake [15] and then stabilized at 5–10% below normal average weight [13]. The energy density of foods and energy-providing beverages on the Swank menus (1.0 kcal/g, 4.2 kJ/g) was lower than the average US diet (1.52 kcal/g, 6.36 kJ/g) [117] which might produce weight loss [118,119]. Increased body mass index (BMI) is a possible risk factor for MS [120] and has been associated with reduced quality of life [121] and increased disability and risk of relapse [122]. Any weight reduction associated with the Swank diet could be beneficial for overweight and obese pwMS but may be contraindicated for individuals with underweight BMI who may be at risk for malnutrition [123]. To assist pwMS who are following the Swank diet in adjusting their energy intake to maintain, lose or gain weight as clinically appropriate, additional guidance on the number of food group servings at various energy levels is needed.

Hyperlipidemia and hypertension are two common comorbidities in pwMS [124]. Swank menu fat composition is consistent with dietary recommendations to reduce saturated fat and increase unsaturated fat [59,97], however, the level and types of unsaturated fat will vary with the quantity and types of oil consumed within the Swank diet guidelines. The DGA recommend the reduction of dietary sodium to < 2300 mg based on data associating increased sodium intake with higher blood pressure [58]. Sodium level of the menus but not the modeled diet exceeded the UL. Modeled diets were lower in sodium because foods without added sodium were used to develop food group nutrient profiles. Top sodium sources on the Swank menus were salt, whole grains, condiments (e.g., mustard, soy sauce, relish) and milk. Reduction in use of added salt and selection of lower sodium condiments could reduce the sodium level of the menus. The Swank diet is also generally consistent with the Dietary Approaches to Stop Hypertension (DASH) guidelines that encourage fruits, vegetables, whole grains, low fat dairy products and sodium < 2300 mg [125].

### 4.3. HEI-2015 and AHEI-2010

Swank menus achieved a high HEI-2015 score of 93.2/100 which is considered a “good” [126] or “A” [67] diet that adheres closely to the DGA. The score is similar to the 87.8–100 scores of exemplary menus used to validate the HEI-2015 [71]. Compared to scores for the US population collected during NHANES 2011–2012 [71], Swank and HEP ranked at the 99th percentile, indicating the menus adhered more closely to the DGA than diets of all but 1% of Americans.

The developers of the AHEI-2010 did not establish cut points to identify “healthy” or “good” diets. However, Swank and HEP diets earned AHEI-2010 scores that fell within the 5th (healthiest) quintile reported by the developers of the AHEI-2010 (AHEI-2010 score > 57.8, median 62.7, for females and > 62.3, median 67.6, for males) [68]. Swank and HEP scores also compared favorably to the AHEI-2010 scores for the third (healthiest) tertile (median, range 64, 59–89) from a study of 7627 British men and women that found associations between lower AHEI-2010 scores and all cause, cardiovascular and cancer mortality [88].

The AHEI-2010 is designed to predict future chronic disease based on current diets of healthy adults. Since pwMS have already been diagnosed with a chronic disease, they be may be taking MS or other medications or have changed their diet. These potential therapeutic changes could affect the predictive ability of AHEI-2010 recommendations in this population. Lower (or worse) AHEI-2010 scores may not be predictive of future chronic disease because current medical advice, medications and diet change could conflict with the recommended quantities for scoring the AHEI-2010 diet.

### 4.4. Limitations

Swank provided only one set of seven-day menus so nutrient amounts were factored to create menus at energy levels appropriate for different adult sex/age groups. These created menus produced uniform differences in nutrient composition, likely a minor limitation. Only one set of menus was assessed so nutrient composition could vary depending on foods selected; however, the modeled diet showed similar nutritional adequacy as menus except for vitamin D which was low on the male model diet but not the menus. Nutritional adequacy of the menus was not assessed for children and pregnant or lactating women. Foods selected by free-living individuals attempting to follow the Swank diet will differ from the menus and may also deviate from the diet guidelines possibly resulting in lower nutritional adequacy. Thus, studies investigating the nutritional adequacy of foods selected by pwMS who are following the Swank diet are warranted. Given the limitations of the study conducted by Swank, randomized controlled studies are needed to investigate the impact of this diet on MS disease course. The clinical trial comparing the effect of the Swank and modified Paleolithic diets [61,62] on fatigue and quality of life will begin to answer some of these questions.

## 5. Conclusions

The Swank diet is one among several dietary regimens promoted for pwMS that are currently under investigation. Swank menus had similar levels of nutritional adequacy for 17 vitamins and minerals as the HEP and had similar high adherence to the DGA. AHEI-2010 scores for Swank and HEP modeled diets for males and females 31-50 years were similar. Swank diet-prescribed supplementation with vitamin E, cod liver oil and a multivitamin/mineral corrected shortfalls in vitamins D and E, folate, calcium and iron but not dietary fiber (males 31–50 years), potassium or choline. Careful selection of foods may be required to meet dietary fiber, potassium and choline requirements and keep sodium intake below the UL. Additional research is needed to assess how well pwMS adhere to this diet pattern, examine the nutritional adequacy of their food selections, and determine impact of the diet on MS disease course.

## Figures and Tables

**Table 1:** Low saturated fat Swank [36,37] diet guidelines.

**Saturated Fat**	≤15 g
**Foods Recommended**	2+ cup-eq ^1^ (~45–250 g) fruit per day, fresh preferred ^2^
	2+ cup-eq (~20–250 g) vegetables ^2^ per day
	4 servings grains per day, whole preferred ^2^
	2 cups (~490 g) dairy products with <1% fat per day
	Protein foods, daily
	Egg whites
	Poultry, white meat, no skin
	White fish and shellfish
	Nuts and seeds ^3^
	4 to 10 teaspoons (20–50 g) oil ^4^ per day
**Foods Limited**	Fatty fish ≤ 50 g (1.75 ounce) per day ^3^
	Whole eggs ≤ 1 per day, ≤ 3 per week
	Caffeinated beverages ≤ 3 cups (237–246 g) per day
	Alcoholic beverages, maximum one drink per day
**Foods Not Recommended**	Beef, pork, dark meat poultry ^5^
	Processed food containing saturated fat
	Butter, animal fats
	High fat dairy productsHigh-sugar products
	Coconut oil, palm oil, margarine, lard, shortening, hydrogenated oil
	Coconut, chocolate, cocoa butter
**Supplements**	1 teaspoon (5 g) cod liver oil
	1 multivitamin/mineral
	1000 mg vitamin C ^2^
	400 IU vitamin E ^2^

^1^ Cup-equivalents = 2 cups raw leafy (~30–140 g), 1 cup raw or cooked (~35–250 g), 1 cup juice (~245–250 g), ½ cup dried (~20–90 g); ^2^ Swank MS Foundation [37] updated these guidelines after *The Multiple Sclerosis Diet Book* [36] was published; ^3^ Unsaturated fat in nuts and seeds and fatty fish are counted as part of the oil allowance; ^4^ 2–4 teaspoons (10–20 g) for weight reduction, 4 teaspoons (20 g) for sedentary individuals, 8–10 teaspoons (40–50 g) for weight gain [36]; ^5^ After one year individuals may consume up to 3 ounces (85 g) per week.

**Table 2:** Mean food group servings of 1719 kcal (7192 kJ) seven-day low saturated fat Swank menus.

Food Group	Mean Servings
**Fruits and Vegetables, Total (cup-equivalents ^1^)**	4.2
**Fruits, Total (cup-equivalents ^1^)**	1.9
Juice	0.4
Whole Fruit	1.4
**Vegetables, Total (cup-equivalents ^1^)**	2.3
Dark-green Vegetables	1.0
Deep-yellow Vegetables	0.2
Tomato	0.3
White Potatoes	0.5
Other Starchy Vegetables	0.1
Other Vegetables	0.5
**Grains, Total (servings ^2^)**	5.8
Whole Grain	4.0
Some Whole Grain	0.0
Refined Grain	1.8
**Meat/Fish/Eggs/Nuts/Seeds, Total (servings ^3^)**	5.5
Beef/Pork/Lamb	0.0
Poultry	1.4
Fish and Shellfish	2.1
Cold Cuts and Sausage	0.0
Organ Meats	0.0
Eggs	0.6
Nuts and Seeds including Butters	1.4
**Dairy and Nondairy, Total (cup-equivalents)**	2.3
Milk, dairy, low fat and fat free	2.3
Yogurt, dairy, fat free	0.1
Milk, non-dairy	0.0
**Fats, Total (servings ^4^)**	4.2
Oil ^5^	3.5
Butter and Other Animal Fats	0.0
Salad Dressing	0.8
**Sweets, Total (servings ^6^)**	6.7

^1^ Serving = 2 cups raw green leafy (~30–140 g), 1 cup raw or cooked fruit/vegetable/juice (~35–250 g), ½ cup (~20–90 g) dried fruit/vegetable; ^2^ Serving = 1 slice bread, ½ cup cooked pasta (70 g), rice (79 g); ^3^ Serving = 1 ounce (28 g) cooked meat/fish/poultry, 1 large whole egg, 2 large egg whites, 1 tablespoon (16 g) peanut butter, ½ ounce (14 g) nuts/seeds; ^4^ Serving = 1 teaspoon (5 g) oil/butter, 15 g mayonnaise; ^5^ does not include unsaturated fat from fatty fish and nuts/seeds; ^6^ Serving = 4 g sugar, ¼ cup (79 g) syrup, 1 tablespoon (20 g) jam.

**Table 3:** Calculated energy, macronutrient and related components ^1^ for seven-day low saturated fat Swank menus without dietary supplements.

Nutrient	Mean	SD ^2^
Energy (kcal)	1719	234
Energy (kJ)	7192	979
Total Protein (g)	79.8	12.9
Total Carbohydrate (g)	256.6	38.6
Total Dietary Fiber (g)	25.8	3.5
Soluble Dietary Fiber (g)	5.7	0.8
Insoluble Dietary Fiber (g)	20.0	2.9
Total Sugars (g)	124.8	23.5
Added Sugars (by Total Sugars) (g)	51.2	25.7
Gluten (g)	11.4	3.4
Glycemic Index (glucose reference)	56.0	3.6
Glycemic Load (glucose reference)	128.9	20.2
Total Fat (g)	47.7	10.4
Total Saturated Fatty Acids (SFA) (g)	8.1	2.0
Total Trans-Fatty Acids (TRANS) (g)	0.2	0.1
Total Monounsaturated Fatty Acids (MUFA) (g)	16.6	3.6
Total Polyunsaturated Fatty Acids (PUFA) (g)	18.8	5.1
Total Conjugated Linoleic Acid (CLA 18:2) (g)	0.0	0.0
PUFA 18:2 (linoleic acid) (g)	16.1	4.6
PUFA 18:3 n-3 (alpha-linolenic acid [ALA]) (g)	2.0	0.6
Omega 6 Fatty Acids (g) ^3^	16.3	4.6
Omega-3 Fatty Acids (g)	2.5	1.0
Omega 6:3 ratio	6.9	1.7
PUFA 20:5 (eicosapentaenoic acid [EPA]) (g)	0.1	0.2
PUFA 22:5 (docosapentaenoic acid [DPA]) (g)	0.1	0.1
PUFA 22:6 (docosahexaenoic acid [DHA]) (g)	0.4	0.7
Cholesterol (mg)	196	141
% Calories from Protein	18.4	2.3
% Calories from Fat	24.1	3.2
% Calories from SFA	4.1	0.7
% Calories from TRANS	0.1	0.0
% Calories from MUFA	8.3	1.2
% Calories from PUFA	9.5	1.8
% Calories from 18:2 linoleic acid ^4^	8.1	1.7
% Calories from 18:3n3 alpha-linolenic acid ^4^	1.0	0.2
% Calories from Carbohydrate	57.5	4.8
% Calories from added sugar ^4^	9.7	4.5
Total Grams (g)	1766	217
Kcal/Gram	1.0	0.1
kJ/Gram	4.2	0.4
Water (g)	1370	168
sodium:potassium ratio	0.70	0.29
calcium:phosphorus ratio	0.72	0.19
calcium:magnesium ratio	2.91	0.86
Phytic Acid (mg/1000 kcal)	577	186
Oxalic Acid (mg/1000 kcal)	200	94
Pantothenic Acid (mg/1000 kcal)	4	0
Betaine (mg/1000 kcal)	111	38

^1^ Calculations were made using multiple decimal places but results are rounded for display purposes; ^2^ SD = standard deviation; ^3^ Omega-6 Fatty Acids = [PUFA 18:2 (linoleic acid) + PUFA 18:3 (linolenic acid)] − PUFA 18:3 *n*3 (alpha-linolenic acid) + PUFA 20:4 (arachidonic acid); ^4^ denominator kcals = (grams protein × 4) + (grams carbohydrate × 4) + (grams fat × 9) + (grams alcohol × 7).

**Table 4:** Percent Recommended Dietary Allowance, Adequate Intake and Tolerable Upper Intake Levels of selected nutrients calculated for the average seven-day Swank low saturated fat menus without dietary supplements.

Sex	Females				Males			
Age, Years	19–30	31–50	51–70	>70	19–30	31–50	51–70	>70
Energy, kcal ^1^	2000	1800	1600	1600	2600	2200	2000	2000
Energy, kJ	8368	7531	6694	6694	10,878	9205	8368	8368
Protein, grams ^2^	93	84	74	74	121	102	93	93
Carbohydrate, grams ^3^	299	269	239	239	388	328	299	299
Fat, grams ^4^	55	50	44	44	72	61	55	55
Saturated Fat, grams ^5^	9	8	8	8	12	10	9	9
**Percent Recommended Dietary Allowance (RDA)**								
Vitamin A, %RDA	161	145	129	129	162	137	125	125
Vitamin C, %RDA	186	167	148	148	201	170	155	155
Vitamin D, %RDA	**91 ^6^**	**82**	**73**	**55**	119	100	**91**	**68**
Vitamin E, %RDA	**78**	**71**	**63**	**63**	102	**86**	**78**	**78**
Vitamin B1, %RDA	165	149	132	132	197	167	152	152
Vitamin B2, %RDA	247	224	202	202	247	228	209	209
Vitamin B3, %RDA	313	282	251	251	356	301	274	274
Vitamin B6, %RDA	184	165	127	127	239	202	140	140
Folate, %RDA	120	108	**96**	**96**	156	132	120	120
Vitamin B12, %RDA	226	204	181	181	294	249	226	226
Calcium, %RDA	131	118	**87**	**87**	170	144	131	109
Copper, %RDA	178	160	142	142	231	196	178	178
Iron, %RDA	**79**	**71**	143	143	232	196	178	178
Magnesium, %RDA	144	126	112	112	145	117	106	106
Phosphorus, %RDA	255	230	204	204	332	281	255	255
Selenium, %RDA	264	237	211	211	343	290	264	264
Zinc, %RDA	139	125	112	112	132	112	101	101
**Average % RDA**	174	157	142	141	215	183	164	161
**MAR, % ^7^**	97	96	95	94	100	99	98	97
**Percent Adequate Intake (AI)**								
Dietary Fiber, %AI	128	108	114	114	103	**87**	100	100
Linoleic Acid, %AI	156	141	136	136	143	121	134	134
α-Linolenic Acid, %AI	207	186	165	165	185	156	142	142
Vitamin K, %AI	309	278	248	248	302	255	232	232
Manganese, %AI	338	304	271	271	344	291	265	265
Choline, %AI	107	**92**	**81**	**81**	102	**86**	**79**	**79**
Potassium, %AI	**87**	**78**	**70**	**70**	113	**96**	**87**	**87**
**Percent Tolerable Upper Intake Level (UL)**								
Sodium, %UL	**118**	**106**	94	94	**153**	**130**	**118**	**118**

^1^ Energy levels were selected from those reported for the Healthy US-Style Eating Pattern [70] and were typically for sedentary individuals [76]; ^2^ For comparison, the protein RDA is 46 g for adult non-pregnant, non-lactating females and 56 g for adult males based on 0.8 g protein per kg of body weight for the reference body weight [65]; ^3^ For comparison, the carbohydrate RDA is 130 g for adult males and adult non-pregnant, non-lactating females [65]; ^4^ For comparison, the Acceptable Macronutrient Distribution Ranges for total fat are 20%–35% energy [65], which is equivalent to 36–62 g, 40–70 g, 44–78 g, 49–86 g and 58–101 g for 1600, 1800, 2000, 2200 and 2600 kcals, respectively; ^5^ The Swank diet recommends ≤ 15 g saturated fat per day. For comparison, the 2015–2020 Dietary Guidelines for Americans [58] recommend < 10% energy from saturated fat which is equivalent to < 18 g, < 20 g, < 22 g, < 24 g and < 29 g for 1600, 1800, 2000, 2200 and 2600 kcals, respectively. The American Heart Association recommends 5–6% energy from saturated fat for individuals who should reduce their low density lipoprotein cholesterol level [59] which is equivalent to 9–11 g, 10–12 g, 11–13 g, 12–15 g and 14–17 g for 1600, 1800, 2000, 2200 and 2600 kcals, respectively; ^6^ Bolded values are below Recommended Dietary Allowance or Adequate Intake or above the Tolerable Upper Intake Level; ^7^ MAR = mean adequacy ratio.

**Table 5:** Nutrient composition and nutritional adequacy of Swank and US-Style Healthy Eating Pattern [70] for males and females 31–50 years modeled using the United States Department of Agriculture Food Group nutrient profiles.

Category	Females 31–50 Years		Males 31–50 Years	
	Swank	HEP ^1^	Swank	HEP
Energy, kcal	1800	1797	2200	2198
Energy, kJ	7531	7519	9205	9196
Protein, grams	80	87	97	100
Protein, %kcal	18	19	18	18
Fat, grams	56	61	68	78
Fat, %kcal	28	31	28	32
Saturated Fat, grams	11	15	13	20
Saturated Fat, %kcal	5	8	5	8
EPA ^2^, grams	0.1	0.1	0.1	0.1
DHA ^3^, grams	0.2	0.2	0.2	0.2
Carbohydrate, grams	254	233	311	286
Carbohydrate, %kcal	57	52	57	52
Dietary Fiber, grams	26	29	32	35
**Percent Recommended Dietary Allowance (RDA)**				
Vitamin A, %RDA	145	125	138	109
Vitamin C, %RDA	195	133	198	141
Vitamin D, %RDA	**42 ^4^**	**45**	**52**	**47**
Vitamin E, %RDA	**74**	**61**	**90**	**74**
Vitamin B1, %RDA	142	153	160	165
Vitamin B2, %RDA	179	185	185	175
Vitamin B3, %RDA	162	160	173	166
Vitamin B6, %RDA	178	274	218	201
Folate, %RDA	161	143	197	172
Vitamin B12, %RDA	288	274	352	304
Calcium, %RDA	115	126	140	134
Copper, %RDA	145	146	177	173
Iron, %RDA	**95**	**91**	261	242
Magnesium, %RDA	120	105	112	**94**
Phosphorus, %RDA	228	239	279	266
Selenium, %RDA	195	193	238	221
Zinc, %RDA	169	171	150	143
**Average %RDA**	155	154	183	166
**MAR, % ^5^**	95	94	97	95
**Percent Adequate Intake (AI)**				
Dietary Fiber, %AI	103	114	**83**	114
Linoleic Acid, %AI	150	143	129	125
α-Linolenic Acid, %AI	195	185	164	157
Vitamin K, %AI	388	147	356	142
Manganese, %AI	266	213	254	199
Choline, %AI	**81**	**77**	**77**	**69**
Potassium, %AI	**67**	**67**	**82**	**79**
**Percent Tolerable Upper Intake Level (UL)**				
Sodium, %UL	66	75	80	84

^1^ US-Style Healthy Eating Pattern; ^2^ Eicosapentaenoic acid; ^3^ Docosahexaenoic acid; ^4^ bolded values are below the RDA or AI; ^5^ MAR = mean adequacy ratio.

## Supplementary Materials

The following are available online at https://www.mdpi.com/2072-6643/11/3/616/s1, Table S1. Food descriptions and amounts used to calculate the nutrient composition of the seven-day low saturated fat Swank menus. Table S2. Healthy Eating Index-2015 (HEI-2015) and Alternate Healthy Eating Index-2010 (AHEI-2010) components. Table S3. Nutrient composition of dietary supplements prescribed for low saturated fat Swank diet. Table S4. Alternate Healthy Eating Index-2010 (AHEI-2010) component and total score for 1800 kcal (7531 kJ) female and 2200 kcal (9205 kJ) male Swank low saturated fat and Healthy US-Style Pattern (HEP) diets.
