# Supplementary material for: Nutrient Composition Comparison between the Low Saturated Fat Swank Diet for Multiple Sclerosis and Healthy U.S.-Style Eating Pattern
Source: Nutrients. 2019 Mar 13;11(3):616. doi: 10.3390/nu11030616 (PMC6470969; doi:10.3390/nu11030616)
Supplement: Supplementary file 1 [file nutrients-11-00616-s001.pdf]

Supplementary Materials:

Table 1. Food descriptions and amounts used to calculate the nutrient composition of the seven-day low saturated fat Swank [1] menus.

Meal	Food Description	Grams [2]	Household Units
------- Sunday/Day 1 -------			
**Breakfast**	Swank Pancakes		4 pancakes
	egg, whole	33.333	
	sugar, white granulated	5.556	
	salt, regular	0.338	
	buttermilk, skim, nonfat or fat free	54.444	
	baking soda	0.511	
	water - tap	3.292	
	flour, white all-purpose, enriched	27.778	
	syrup, pancake syrup, maple-flavored, regular	78.750	4 TB [3]
	eggs, poached	50.000	1 unknown size
	juice not specified: orange, juice, unknown type	249.000	1 CP [4], without ice
**Morning Snack**	milk, skim	245.000	8 FO [5]
	crackers, Ry-Krisp Natural	28.350	1 Oz [6]
**Lunch**	Swank Pasta Salad		1 serving (1/6 recipe)
	broccoli, cooked from fresh	28.600	
	cauliflower, cooked from fresh	32.240	
	rotini, whole wheat, cooked in unsalted water	103.948	
	celery, raw	16.833	
	peas, green peas, cooked from frozen	21.067	
	tomato, raw	30.000	
	pimento	18.900	
	garlic, fresh	0.500	
	mayonnaise or mayo type dressing, real, regular, commercial	36.667	
	vinegar, red wine	2.490	
	salt, regular	1.014	
	basil - fresh	0.074	
	pepper (ground), black	0.100	
	crackers, saltine or soda, whole wheat	7.500	2.5 each - 2" [7] square
	milk, skim	245.000	8 FO
**Afternoon Snack**	fresh fruit: apple, fresh, with skin	182.000	1 medium - 3" diameter
	Swank Nuts	13.748	1 serving
	peanuts, roasted, oil roasted, salted	3.248	3.67 nuts
	almonds, roasted, oil roasted, salted	4.725	3.67 nuts
	cashews, roasted, oil roasted, salted	5.775	3.67 nuts
**Dinner**	Teriyaki Chicken		1 serving (1/5 recipe)
	chicken, breast, skin removed before cooking	81.000	
	soy sauce, regular	19.581	
	Stir-Fried Vegetables		1 serving (1/4 recipe)
	broccoli, cooked from frozen	19.975	
	cauliflower, cooked from frozen	20.872	
	brussels sprouts, cooked from frozen	21.546	
	oil, not specified	3.405	1/4 TB
	oil, soybean - unhydrogenated	1.861	
	oil, canola (canbra or rapeseed)	0.773	

Meal	Food Description	Grams [2]	Household Units
	oil, olive	0.139	
	oil, corn	0.351	
	oil, sunflower	0.105	
	oil, cottonseed	0.140	
	oil, peanut	0.035	
	salt, regular	0.760	
	garlic, fresh	0.750	
	rice, brown, unknown cooking type, cooked in unsalted water	101.000	1/2 CP, after cooking
	sorbet, fruit	106.000	1/2 CP
-- **Monday/Day 2** --			
**Breakfast**	Swank Banana Strawberry Drink		1 serving (1/2 recipe)
	milk, skim	122.500	
	banana, fresh or ripe	59.000	
	strawberries, frozen, unsweetened	55.250	
	wheat, germ, crude	3.594	
	vanilla extract	2.167	
	wheat bread, whole wheat, regular, commercial	36.000	1 medium slice
	peanut butter, natural, unknown if salted	10.667	2 TS [8]
**Morning Snack**	milk, skim	245.000	8 FO
**Lunch**	Turkey Sandwich		1 sandwich
	wheat bread, whole wheat, regular, commercial	72.000	2 medium slice
	turkey, light meat - unknown part, skin removed before cooking	85.000	85 G [9], after cooking, edible portion
	mayonnaise or mayo type dressing, real, regular, commercial	9.167	2 TS
	fresh fruit: grapes, fresh	151.000	1 CP
	milk, skim	245.000	8 FO
	Swank Chewy Oatmeal Cookies		2 cookies (2/72 recipe)
	egg, whole	2.778	
	oil, not specified (see Sunday/Day 1 Dinner)	4.036	
	sugar, white granulated	5.556	
	sugar, brown sugar	6.111	
	vanilla extract	0.120	
	salt, regular	0.084	
	baking soda	0.128	
	water - tap	1.234	
	flour, white all-purpose, enriched	6.944	
	oatmeal - dry, regular or quick	4.500	
	raisins, uncooked	2.014	
	walnuts (half recipe amount)	1.625	
**PM Snack**	Swank Nuts (see menu Sunday/Day 1 Afternoon Snack)	13.748	1 serving
**Dinner**	Swank Easy Salmon Patties		2 patties (2/6 recipe)
	salmon, canned - drained, pink, with salt	46.682	
	egg, whole	16.667	
	crackers, butter, regular, regular	18.000	
	scallions or spring onions, raw (tops and bulb)	5.000	
	dressing for salads, mayonnaise or mayo type dressing, real, regular, commercial	9.167	
	tarragon (ground)	0.267	
	oil, unspecified (see Sunday/Day 1 Dinner)	5.458	
	Tossed Salad		2 CP
	carrots, raw	24.400	

Meal	Food Description	Grams [2]	Household Units
	tomato, raw	36.000	
	lettuce, iceberg	17.600	
	endive (curly), raw	17.600	
	spinach, raw	17.600	
	lettuce, romaine or cos	17.600	
	lettuce, green leaf	17.600	
	Swank French Dressing		2 TS (2/48 recipe)
	mustard, regular, regular	0.108	
	sugar, white granulated	0.521	
	salt, regular	0.127	
	oil, unspecified (see Sunday/Day 1 Dinner)	4.539	
	vinegar, distilled	1.240	
	lemon juice, fresh	1.271	
	celery juice (substituted for onion juice)	0.205	
	paprika	0.094	
	catsup, regular	0.625	
	potato, baked, plain, skin eaten	173.000	1 medium - 2 1/4" to 3" diameter
	fresh fruit: watermelon, fresh	152.000	1 CP, cubed
**--- Tuesday/Day 3 ---**			
**Breakfast**	Swank Granola		1/2 CP (1/36 recipe)
	oatmeal - dry, regular or quick	11.250	
	wheat, bran (unprocessed)	3.222	
	cashews, roasted, oil roasted, salted	1.792	
	almonds, raw (dried - unblanched)	1.806	
	sunflower seeds, raw (dried)	1.944	
	sesame seeds, kernels (hulled), toasted kernels, salted	1.778	
	wheat, germ, crude	6.389	
	milk, unprepared dry powder, nonfat, regular	1.889	
	salt, regular	0.507	
	oil, unspecified (see Sunday/Day 1 Dinner)	6.052	
	water - tap	6.583	
	honey	14.125	
	vanilla extract	1.083	
	raisins, uncooked	3.021	
	cranberries, dried	2.833	
	juice: apple, juice or cider, unknown type, unknown type	248.000	1 CP, without ice
**AM Snack**	fresh fruit: strawberries, fresh	152.000	1 CP, halves
**Lunch**	Tuna Salad Sandwich (modified from default sandwich)		1 sandwich
	wheat bread, whole wheat, regular, commercial	72.000	2 medium slices
	lettuce, iceberg	10.000	
	tuna, canned, light, water pack, regular, drained - not rinsed	66.519	
	celery, raw	21.813	
	mayonnaise or mayo type dressing, real, regular, commercial	9.167	2 TS
	pickle relish, sweet	6.614	
	salt, regular	0.328	
	fresh fruit: banana, fresh or ripe	118.000	1 medium - 7" to 7 7/8" long
	milk, skim	245.000	8 FO
**PM Snack**	Swank Nuts (see menu Sunday/Day 1 Afternoon Snack)	13.748	1 serving
**Dinner**	Swank Easy Oven Chicken		1 serving (1/3 recipe)
	chicken, breast, skin removed before cooking	80.000	

Meal	Food Description	Grams [2]	Household Units
	basil, ground	0.500	
	pepper (ground), black	0.383	
	paprika	0.024	
	Worcestershire sauce, regular	1.910	
	rice - uncooked, brown	7.708	
	potato, baked, plain, skin eaten	99.667	
	tomato, cooked from fresh	37.000	
	peppers, green pepper - sweet, cooked	6.400	
	mushrooms, canned - drained, regular	37.799	
	Tossed Salad (see Monday/Day 2 Dinner)	151.600	2 CP
	Swank Omar's Special Dressing		2 TS (2/120 recipe)
	garlic, fresh	0.050	
	egg - raw, whole	0.833	
	sugar, white granulated	0.069	
	salt, regular	0.051	
	paprika	0.009	
	mustard, powder	0.006	
	Worcestershire sauce, regular	0.048	
	catsup, regular	1.000	
	vinegar, distilled	0.992	
	oil, unspecified (see Sunday/Day 1 Dinner)	3.631	
	water - tap	1.317	
	Swank Angel Food Cake		1 serving (1/8 recipe)
	eggs, white only, cooked	30.563	
	salt, regular	0.380	
	cream of tartar	0.375	
	sugar, white granulated	37.500	
	flour, cake or pastry	21.406	
	vanilla extract	0.271	
	almond extract	0.313	
	lemon extract	0.156	
**-- Wednesday/Day 4 --**			
**Breakfast**	wheat bread, whole wheat, regular, commercial	36.000	1 medium slice
	peanut butter, natural, unknown if salted	10.667	2 TS
	cereal, ready-to-eat, unknown type (Cheerios)	28.000	1 CP
	milk, skim	122.500	1/2 CP
	juice: orange, juice, unknown type	249.000	1 CP, without ice
**Morning Snack**	milk, skim	245.000	8 FO
	chocolate sauce, syrup (thin), nonfat	17.500	1 TB
**Lunch**	Peanut Butter and Jelly Sandwich		1 sandwich
	wheat bread, whole wheat, regular, commercial	72.000	2 medium slices
	peanut butter, natural, unknown if salted	21.333	4 TS
	jelly, regular	14.000	14 G
	fresh fruit: banana, fresh or ripe	118.000	1 medium - 7" to 7 7/8" long
	milk, skim	245.000	8 FO
**Afternoon Snack**	Swank Nuts (see Sunday/Day 1 Afternoon Snack)	13.748	1 serving
**Dinner**	White Fish with Beer Batter		1 serving
	fish and seafood, whitefish, cooked from fresh or frozen	154.000	
	flour, white all-purpose, enriched	6.845	
	salt, regular	1.322	

Meal	Food Description	Grams [2]	Household Units
	oil, unspecified (see Sunday/Day 1 Dinner)	3.946	
	Swank Oven-Fried Potatoes		1 serving (1/2 recipe)
	potato, baked, plain, skin not eaten	131.000	1 medium
	oil, unspecified (see Sunday/Day 1 Dinner)	6.809	1/2 TB
	salt, regular	0.400	1 dash
	Tossed Salad (see Monday/Day 2 Dinner)	151.600	2 CP
	lemon juice, unknown type	15.250	1 TB
	fresh fruit: apple, fresh, with skin	182.000	1 medium - 3" diameter
**--- Thursday/Day 5 ---**			
**Breakfast**	eggs, poached	50.000	1 unknown size
	wheat bread, whole wheat, regular, commercial	36.000	1 medium slice
	jams or preserves, regular	20.000	1 TB
	fresh fruit: orange, fresh	131.000	1 medium - 2 5/8" diameter
**Morning Snack**	milk, skim	245.000	8 FO
**Lunch**	Swank Easy Vegetable Soup		1 serving (1/9 recipe)
	water - tap	105.333	
	bouillon - unprepared (cube or granules), regular	1.556	
	potato, boiled, with skin	49.400	
	carrots, cooked from fresh	24.960	
	celery, cooked	24.333	
	peppers, green pepper - sweet, cooked	3.167	
	onion, white, yellow or red, cooked	22.750	
	corn, yellow, canned, regular - drained	25.200	
	tomato, canned, regular, plain	25.200	
	tomato, cooked from fresh	15.733	
	rolls, whole wheat	28.000	1 small - 2" square, pan/dinner roll
	fresh fruit: apple, fresh, with skin	182.000	1 medium - 3" diameter
	Swank Chocolate Brownies		1 serving (1/16 recipe)
	egg, whole	6.250	
	sugar, white granulated	9.375	
	vanilla extract	0.135	
	chocolate sauce, syrup (thin), nonfat	8.750	
	oil, unspecified (see Day 1 Dinner)	4.539	
	flour, white all-purpose, enriched	5.859	
	salt, regular	0.190	
	Walnuts (half recipe amount)	3.656	
**Afternoon Snack**	Swank Nut (see Sunday/Day 1 Afternoon Snack)	13.748	1 serving
**Dinner**	Teriyaki Shrimp		1 serving (1/3.5 recipe)
	shrimp, cooked from fresh	78.523	
	sauce, teriyaki sauce, commercial	21.438	
	vegetables, mixed and/or combination vegetables, Oriental (broccoli/mushroom/water chestnut/red pepper)	127.000	1 CP
	rice, brown, unknown cooking type, cooked in unsalted water	101.000	1/2 CP, after cooking
	Swank Fresh Apple Cake		1 serving (1/12 recipe)
	sugar, white granulated	16.667	
	apple, baked or scalloped, with skin, unsweetened	14.999	
	flour, cake or pastry	17.125	
	baking soda	0.383	
	salt, regular	0.253	
	cinnamon (ground)	0.217	

Meal	Food Description	Grams [2]	Household Units
	nutmeg	0.097	
	allspice (ground)	0.083	
	oil, unspecified (see Sunday/Day 1 Dinner)	4.539	
	egg, whole	4.167	
	margarine, spread, 60% fat, salted, unknown type of oil	0.298	
	raisins, uncooked	6.042	
	walnuts (half recipe amount)	2.438	
-- **Friday/Day 6** --			
**Breakfast**	bagel, whole wheat, plain or with seasoning	105.000	1 medium - 3 1/2" - 4" diameter
	Swank Yogurt Cream Cheese		2 servings (2/44 recipe)
	yogurt, plain, nonfat (<1% fat)	89.091	
	water, lost in evaporation process	-57.955	
	Fresh Fruit: Mixed	163.455	1 serving
	cantaloupe, fresh	42.720	
	blueberries, fresh	29.600	
	pear, fresh	32.200	
	pineapple, fresh	33.000	
	mandarin orange, fresh	25.935	
	milk, skim	245.000	8 FO
**Morning Snack**	milk, skim	245.000	8 FO
**Lunch**	Tossed Salad (see Monday/Day 2 Dinner)	151.600	2 CP
	Swank French Dressing (see Monday/Day 2 Dinner)		4 TS (4/48 recipe)
	rolls, whole wheat	28.000	1 small - 2" square, pan/dinner roll
	milk, skim	245.000	8 FO
	Swank Drop Sugar Cookies		2 cookies (2/24 recipe)
	egg, whole	8.333	
	oil, unspecified (see Sunday/Day 1 Dinner)	12.106	
	vanilla extract	0.722	
	sugar, white granulated	12.500	
	flour, white all-purpose, enriched	23.438	
	baking powder, regular	0.767	
	salt, regular	0.253	
**Afternoon Snack**	fresh fruit: banana, fresh or ripe	118.000	1 medium - 7" to 7 7/8" long
**Dinner**	spaghetti noodles, whole wheat, cooked in unsalted water	70.000	1/2 CP
	Swank Meatless Spaghetti Sauce		1 serving (1/10 recipe)
	onion, white, yellow or red, cooked	13.650	
	garlic, fresh	0.900	
	oil, olive	2.700	
	salt, regular	0.608	
	pepper (ground), black	0.100	
	oregano, ground	0.180	
	basil, ground	0.150	
	cayenne pepper	0.022	
	peppers, green pepper - sweet, cooked	5.700	
	paste, tomato	34.019	
	sauce, tomato, regular	79.379	
	wine, cabernet sauvignon	11.760	
	sugar, brown sugar	1.375	
	mushrooms, cooked from fresh	10.800	
	olives, black	45.359	

Meal	Food Description	Grams [2]	Household Units
	nutrients, primary energy sources, alcohol	-1.247	
	nutrients, primary energy sources, energy (kilocalories)	0.000	
	Tossed Salad (see menu Monday/Day 2 dinner)	151.600	2 CP
	Swank Russian Dressing [10]		2 TS (2/120 recipe)
	sauce, tomato, regular	3.780	
	vinegar, distilled	1.983	
	salt, regular	0.203	
	paprika	0.019	
	sauce, Worcestershire sauce, regular	0.286	
	oil, unspecified (see Day 1 Dinner)	3.631	
	mustard, powder	0.013	
	garlic, powder	0.027	
	onion, white, yellow or red, raw	1.167	
	sugar, white granulated	0.069	
	French bread	32.000	1 small slice - 2" x 2 1/2" x 1 3/4"
	Swank Fresh Raspberry Sherbet		1 serving (1/16 recipe)
	gelatin powder, plain	0.438	
	sugar, white granulated	9.375	
	water - tap	29.625	
	raspberries, fresh, red	30.750	
	juice or flavored drink, lemon juice, fresh	1.906	
	egg - raw, white only	4.175	
	salt, regular	0.048	
**-- Saturday/Day 7 --**			
**Breakfast**	Swank Waffles (Whole Wheat)		1 serving (1/6 recipe)
	flour, whole wheat, use in recipes not containing yeast	40.000	
	baking powder, regular	3.067	
	salt, regular	1.014	
	sugar, white granulated	2.083	
	milk, skim, nonfat or fat free	51.042	
	egg, whole	8.333	
	eggs, white only, cooked	11.133	
	oil, unspecified (see Sunday/Day 1 Dinner)	4.539	
	fresh fruit: peach, fresh	150.000	1 medium - 2 2/3" diameter
	milk, skim	245.000	8 FO
**Morning Snack**	Swank Pumpkin Bread		1 serving (1/24 recipe)
	flour, white all-purpose, enriched	13.021	
	baking soda	0.383	
	salt, regular	0.127	
	cinnamon (ground)	0.054	
	cloves (ground)	0.045	
	sugar, white granulated	16.667	
	pumpkin, canned	18.900	
	oil, unspecified (see Day 1 Dinner)	2.270	
	walnuts (half recipe amount)	1.219	
	dates	3.063	
	milk, skim	245.000	8 FO
**Lunch**	pita bread, whole wheat	57.000	1 medium - 6" diameter
	salad, chicken ("mayo" dressing), plain	75.333	1/3 CP
	chicken, light meat - unknown part, skin removed before cooking	39.649	

Meal	Food Description	Grams [2]	Household Units
	celery, raw	14.832	
	mayonnaise or mayo type dressing, real, nonfat	18.797	
	relish, pickle relish, sweet	4.497	
	fresh fruit: apple, fresh, with skin	182.000	1 medium - 3" diameter
	milk, skim	245.000	8 FO
**Afternoon Snack**	Swank Nuts (see menu day 1 afternoon snack)	13.748	1 serving
**Dinner**	Swank Tuna Pot Pie		1 serving (1/6 recipe)
	oil, unspecified (see Day 1 Dinner)	9.079	
	flour, white all-purpose, enriched	6.944	
	salt, regular	1.521	
	pepper (ground), black	0.096	
	marjoram (dried)	0.024	
	milk, skim, nonfat or fat free	68.056	
	tuna, canned, light, water pack, regular, drained - not rinsed	64.495	
	mixed and/or combination vegetables, peas and carrots, cooked from frozen	46.400	
	onion, pearl, cooked	24.513	
	sauce, Worcestershire sauce, regular	1.910	
	flour, white all-purpose, enriched	20.833	
	salt, regular	0.760	
	oil, unspecified (see Sunday/Day 1 Dinner)	9.079	
	milk, skim, nonfat or fat free	5.104	
	Tossed Salad (see menu Monday/Day 2 Dinner)	151.600	2 CP
	Swank Omar's Special Dressing (see Tuesday/Day 3 Dinner)		2 TS (2/120 recipe)
	rolls, whole wheat	28.000	1 small - 2" square, pan/dinner roll
	Swank Apple Crisp		1 serving (1/6 recipe)
	apple, baked or scalloped, with skin, unsweetened	96.255	
	cinnamon (ground)	0.325	
	nutmeg	0.292	
	sugar, white granulated	11.111	
	oatmeal - dry, regular or quick	13.500	
	flour, white all-purpose, enriched	10.417	
	sugar, brown sugar	36.667	
	oil, unspecified (see Sunday/Day 1 Dinner)	6.809	

[1] adapted from menus and recipes in: Swank RL, Dugan B.B. *The Multiple Sclerosis Diet Book. A Low-Fat Diet for the Treatment of M.S.* New York, New York, USA: Doubleday; 1987; [2] weights from the Minnesota Nutrition Data System for Research *NDSR* [computer program]. Version 2017. Minneapolis, MN, USA: Nutrition Coordinating Center; 2017. Values are unrounded and are not meant to imply this level of accuracy. Weights for cooked foods are the weight after cooking, edible portion. [3] TB = tablespoon; [4] CP = cup; [5] FO = fluid ounce; [6] OZ = ounce; [7] " = inches; [8] TS = teaspoon; [9] G = grams; [10] used 2 teaspoons dressing, however, menu listed serving size as 3 teaspoons

Table S2. Healthy Eating Index-2015 (HEI-2015) and Alternate Healthy Eating Index-2010 (AHEI-2010) components.

**Components**	**HEI-2015 (100 maximum points)**	**AHEI-2010 (110 maximum points)**
Adequacy		
Vegetables		
- Vegetables, total	c-eq [1]/1000 kcal including potatoes and legumes (5)	servings [2]/day excluding potatoes and legumes (10)
- Vegetables, greens and beans	c-eq/1000 kcal (5)	not included as separate component
Fruits		
- Fruit, total (i.e., includes juice)	c-eq/1000 kcal (5)	not included
- Fruit, whole (i.e., excludes juice)	c-eq/1000 kcal (5)	servings [3]/day (10)
Grains		
- Grains, whole	oz-eq [4]/1000 kcal (10)	grams whole grains/day [5] (10)
Dairy	c-eq/1000 kcal (10)	not included
Protein Foods		
- Protein Foods, total	oz-eq/1000 kcal (5)	not included
- Protein Foods, seafood and plants	oz-eq/1000 kcal (5)	not included
- Protein Foods, nuts and legumes	not included as separate component	servings/day (10)
Fats		
- Fatty Acids	(PUFA [6] + MUFA [7])/SFA [8] (10)	PUFA, % energy (10), excluding EPA [9] + DHA [10]
- Long chain omega-3 (EPA + DHA)	not included as separate component	mg/day (10)
Moderation [11]		
- Grains, refined	oz-eq/1000 kcal (10)	not included
- Protein Foods, red and processed meat	not included as separate component	servings/day (10)
- Fats, saturated fat	% energy (10)	not included
- Fats, *trans*-fat	not included as separate component	% energy (10)
- Added sugars	% energy (10)	not included
- Sugar-sweetened beverages and fruit juice	not included as separate component	servings/day (10)
- Sodium	g/1000 kcal (10)	mg/day [5, 12] (10)
- Alcohol	not included [13]	drinks/day [5, 14] (10)

[1] c-eq = cup-equivalent, 1 cup raw, cooked or juice, 2 cups raw leafy, ½ cup dried; [2] serving = ½ cup or 1 cup raw, leafy; [3] serving = 1 piece fruit or ½ cup berries; [4] oz-eq = ounce-equivalent, 1 slice bread, ½ cup cooked pasta (70 g) or rice (79 g); [5] criteria for minimum and maximum score varies with sex; [6] PUFA = polyunsaturated fatty acids; [7] MUFA = monounsaturated fatty acids; [8] SFA = saturated fatty acids; [9] EPA = Eicosapentaenoic acid; [10] DHA = Docosahexaenoic acid; [11] moderation components are reverse-scored, i.e., higher intake receives a lower score; [12] Minimum and maximum scores for males and females were to be assigned based on the dataset's lowest and highest decile of sodium intake for each sex but the menu modeling did not provide a sodium distribution; therefore, 10th and 90th percentiles from the 2007-2008 NHANES for 20+ year old males and females were used instead; [13] alcohol is not a separate component but energy from alcohol is included in total energy intake which is used to compute scores that are calculated per 1000 kcals or as % energy; [14] no alcohol intake receives 2.5 points.

Table S3. Nutrient composition [1] of dietary supplements prescribed for low saturated fat Swank diet.

Nutrient	Females 19-50 years	Females ≥51 years	Males 19-50 years	Males ≥51 years
Energy (kcal)	45	45	45	45
Total Protein (g)	0	0	0	0
Total Fat (g)	5	5	5	5
Total Carbohydrate (g)	0	0	0	0
Total Saturated Fatty Acids (SFA) (g)	1	1	1	1
Total Monounsaturated Fatty Acids (MUFA) (g)	0	0	0	0
Total Polyunsaturated Fatty Acids (PUFA) (g)	0	0	0	0
Omega-3 Fatty Acids in Supplements (mg)	1100	1100	1100	1100
Cholesterol (mg)	20	20	20	20
PUFA 20:5 (eicosapentaenoic acid [EPA]) (g)	0.4	0.4	0.4	0.4
PUFA 22:6 (docosahexaenoic acid [DHA]) (g)	0.5	0.5	0.5	0.5
Total Vitamin A Activity (International Units) (IU)	3350	4350	4350	4350
Vitamin D (calciferol) (mcg)	35	35	27.5	27.5
Vitamin E (International Units) (IU)	432.5	440	432.5	435.5
Vitamin K (phylloquinone) (mcg)	25	20	20	20
Vitamin C (ascorbic acid) (mg)	1060	1120	1060	1120
Thiamin (vitamin B1) (mg)	1.5	4.5	1.35	4.5
Riboflavin (vitamin B2) (mg)	1.7	3.4	1.7	3.4
Niacin (vitamin B3) (mg)	20	20	18	20
Pantothenic Acid (mg)	10	15	16	15
Vitamin B-6 (mg)	2	6	3	6
Synthetic Folate (folic acid) (mcg)	400	400	400	400
Vitamin B-12 (cobalamin) (mcg)	6	25	18	25
Biotin (mcg)	300	30	75	30
Lycopene (mcg)	0	0	300	370
Calcium (mg)	500	500	210	120
Chromium (mcg)	120	180	120	180
Copper (mg)	2	2.2	2	2.2
Iodine (mcg)	150	150	0	150
Iron (mg)	18	0	0	0
Magnesium (mg)	50	50	140	110
Manganese (mg)	2	4.2	2	4.2
Molybdenum (mcg)	0	90	0	90
Phosphorus (mg)	0	0	0	0
Potassium (mg)	0	0	0	0
Selenium (mcg)	20	27	110	117
Sodium (mg)	0	0	0	0
Zinc (mg)	15	24	15	24
Total Dietary Fiber (g)	0	0	0	0
Soluble Dietary Fiber (g)	0	0	0	0
Insoluble Dietary Fiber (g)	0	0	0	0
Betaine (mg)	0	0	0	0
Choline (mg)	0	0	0	0
Caffeine (mg)	0	0	0	0
Phytic Acid (mg)	0	0	0	0
Total Trans-Fatty Acids (TRANS) (g)	0	0	0	0
PUFA 18:3 n-3 (alpha-linolenic acid [ALA]) (g)	0	0	0	0

[1] Obtained from Nutrition Data System for Research (NDSR) software version 2017, developed by the Nutrition Coordinating Center (NCC), University of Minnesota, Minneapolis, Minnesota, USA (May 2017).

Table S4. Alternate Healthy Eating Index-2010 (AHEI-2010) component and total score for 1800 kcal (7531 kJ) female and 2200 kcal (9205 kJ) male Swank low saturated fat and Healthy US-Style Pattern (HEP) [1] diets.

	**1800 kcal (7531 kJ) Female 31-50 years**		**2200 kcal (9205 kJ) Male 31-50 years**	
**Component (maximum score)**	**Swank**	**HEP[1]**	**Swank**	**HEP**
	------------------------- Score -------------------------			
Vegetables (10)	4.4	6.9	5.3	8.1
Fruit (10)	7.4	5.3	9.1	7.1
Whole grains (10)	8.9	6.4	9.0	6.2
Sugar-sweetened beverages and fruit juice (10)	5.4	5.6	4.4	4.1
Nuts and legumes (10)	10.0	10.0	10.0	10.0
Red/processed meat (10)	10.0	4.9	10.0	3.8
Trans-fat (10)	10.0	10.0	10.0	10.0
Long chain omega-3 fatty acids [2] (10)	10.0	8.8	10.0	10.0
PUFA [3] (10)	10.0	9.7	10.0	9.8
Sodium (10)	9.2	8.5	10.0	9.9
Alcohol (10)	2.5	2.5	2.5	2.5
Total Score (110)	87.7	78.6	90.3	81.5

[1] Healthy US-Style Eating Pattern; [2] Eicosapentaenoic acid (EPA) and Docosahexaenoic acid (DHA); [3] Polyunsaturated Fatty Acids excluding EPA and DHA.
